# Plant Products for Pharmacology: Application of Enzymes in Their Transformations

**DOI:** 10.3390/ijms9122447

**Published:** 2008-12-04

**Authors:** Marie Zarevúcka, Zdeněk Wimmer

**Affiliations:** 1 Institute of Organic Chemistry and Biochemistry, AS CR, Flemingovo náměstí 2, 166 10 Prague 6 – Dejvice, Czech Republic. E-Mail: zarevucka@uochb.cas.cz; 2 Institute of Experimental Botany AS CR, Isotope Laboratory, Vídeňská 1083, 142 20 Prague 4 – Krč, Czech Republic

**Keywords:** Lipase, hydrolysis, esterification, plant oil, phytosterol, supercritical fluid, ionic liquid

## Abstract

Different plant products have been subjected to detailed investigations due to their increasing importance for improving human health. Plants are sources of many groups of natural products, of which large number of new compounds has already displayed their high impact in human medicine. This review deals with the natural products which may be found dissolved in lipid phase (phytosterols, vitamins etc.). Often subsequent convenient transformation of natural products may further improve the pharmacological properties of new potential medicaments based on natural products. To respect basic principles of sustainable and green procedures, enzymes are often employed as efficient natural catalysts in such plant product transformations. Transformations of lipids and other natural products under the conditions of enzyme catalysis show increasing importance in environmentally safe and sustainable production of pharmacologically important compounds. In this review, attention is focused on lipases, efficient and convenient biocatalysts for the enantio- and regioselective formation / hydrolysis of ester bond in a wide variety of both natural and unnatural substrates, including plant products, eg. plant oils and other natural lipid phase compounds. The application of enzymes for preparation of acylglycerols and transformation of other natural products provides big advantage in comparison with employing of conventional chemical methods: Increased selectivity, higher product purity and quality, energy conservation, elimination of heavy metal catalysts, and sustainability of the employed processes, which are catalyzed by enzymes. Two general procedures are used in the transformation of lipid-like natural products: (a) Hydrolysis/alcoholysis of triacylglycerols and (b) esterification of glycerol. The reactions can be performed under conventional conditions or in supercritical fluids/ionic liquids. Enzyme-catalyzed reactions in supercritical fluids combine the advantages of biocatalysts (substrate specificity under mild reaction conditions) and supercritical fluids (high mass-transfer rate, easy separation of reaction products from the solvent, environmental benefits based on excluding organic solvents from the production process).

## 1. Introduction

**Scheme 1.**
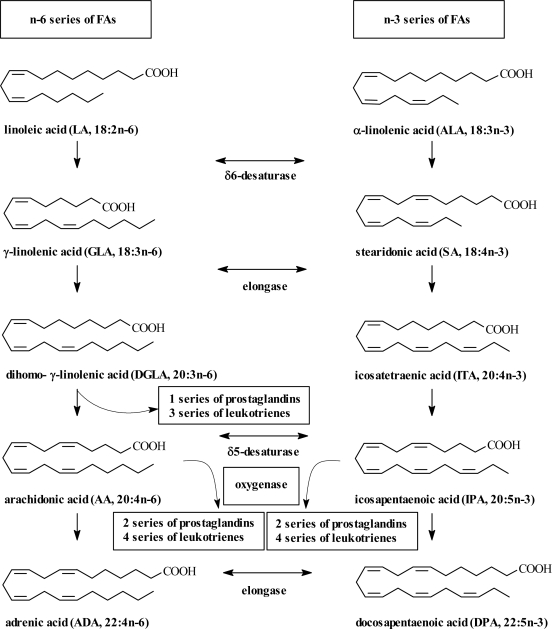
Metabolic pathways of polyunsaturated fatty acids.

Plant products with low polarity are mainly understood to be represented by plant lipids, formed by glycerides, important sources of fatty acids (FAs), and by compounds soluble in lipid phase (phytosterols, vitamins etc.). Glycerides are important natural and sustainable sources of FAs. Among them, ω-3 and ω-6 polyunsaturated fatty acids (PUFAs) belong among the main FA classes, derived biosynthetically from linoleic acid (LA) (Scheme 1). LA and ALA are termed essential fatty acids (EFAs) because mammalian cells are unable to synthesize these compounds *de novo*. LA can be converted sequentially by the known biosynthetic pathways into the C_18_ ω-3 and ω-6 fatty acids, α-linolenic acid (ALA) and γ-linolenic acid (GLA), and into the C_20_ – arachidonic acid (AA) and dihomo-γ-linolenic acid (DGLA). ALA is converted into longer chain ω-3 fatty acids, such as C_20_ – icosapentaenoic (eicosapentaenoic) acid (EPA) and C_22_ – docosahexaenoic acid (DHA). Increasing evidence indicates that even if LA and ALA can be converted into their longer chain length metabolites, the conversion rate in humans is very slow, resulting in an estimated 2 to 10% of ALA being converted to DHA or EPA, and may be negatively affected by increasing age and any health injury [1,2]. This finding is a basis for a general suggestion that EFAs are likely to be dietary additives. This view may be supported by the experimental data on elevation of the cellular levels of both these ω-3 PUFAs after supplying fish oil to humans [3]. ω-3 PUFAs are of particular interest from a nutritional standpoint since the intake of these fatty acids is considered to be low in Western diets [4]. They have been investigated for their cardioprotective and anti-inflammatory roles, a treatment of certain forms of mental illness and as major targets of psychoactive medications, which has resulted in suggesting their increase as dietary supplements [5–9]. PUFAs may be directly administered as free fatty acids (FFAs), ethyl esters (FAEEs) or triacylglycerols (TAG). FFAs seem to be the best absorbable pharmaceutical form for preventing cardiovascular diseases [10] and FAEEs are used for treating pancreatic cancer cachexia (associated chronic weight loss) [11].

## 2. PUFA Sources

Natural sources of n-6 PUFAs contain variable amounts of these acids rarely exceeding 25% GLA. Oils produced from borage, evening primrose and blackcurrant seeds are rich sources of GLA and contain 17–25%, 8–10% and 10–12% GLA, respectively [12–14]. Thus, there has always been a pharmacologically motivated increasing interest in producing higher concentrates of GLA. Different techniques have been developed to enrich GLA from natural sources, and they include urea fractionation of FAs [15–17], separation on Y-zeolite and lipase-catalyzed reactions, namely selective hydrolysis of GLA-containing triacylglycerols [18], and selective esterification of GLA-containing FA mixtures [19]. The only commercial sources of n-3 PUFAs (EPA and DHA) and AA are fish oils and animal viscera [20, 21]. However, many microalgae species, including krill, have also been found to be rich in oils containing various amounts of PUFAs [22–25]. Marine fish oils contain substantial amounts of PUFAs, and are currently the major sources of EPA and DHA [26]. Many plant sources are composed of essential long-chain fatty acids (LCFAs) such as oleic acid (C18:1), linoleic acid (C18:2) and linolenic acid (C18:3). Palm olein fractions contain a high proportion of unsaturated fatty acids, in particular C18:1 (63.1%) and C18:2 (23.9%) [27]. These LCFAs are generally recognized as useful nutrient substrates.

## 3. Triacylglycerol structure

**Figure 1.**
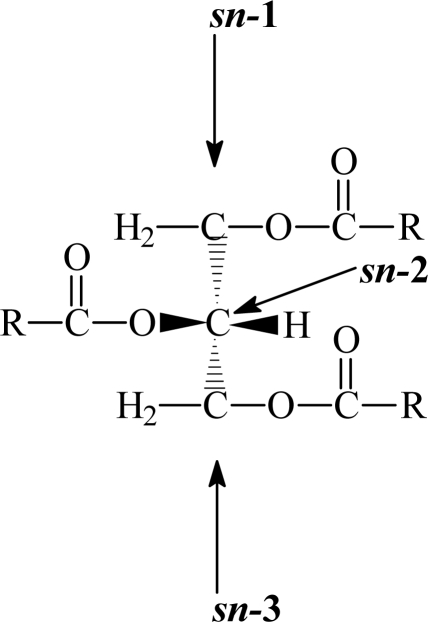
Triacylglycetol structure, R = fatty acid acyl chains.

In nearly all plant cells, energy reserves in the forms of FAs are contained in a triacylglycerol molecule. Since the glycerol molecule does not have rotational symmetry, all the carbon atoms distinguish from each other. They are classified by the *sn* (stereochemical numbering) system (*sn*-1, *sn*-2, and *sn*-3; Figure 1) according to the recommendation of the IUPAC-IUB Commission of Biochemical Nomenclature. The plant triacylglycerols can posses a great number of different fatty acids, although eight particular ones account for some 97% of those present in commercial vegetable oils. The seed triacylglycerols are usually characterized by predominance of C_18_-unsaturated acids and PUFAs, and this distinguishes them from animal fats, which are generally of a more saturated nature. The C_18_-unsaturated fatty acids (oleic, linoleic, and linolenic) are particularly important and govern, to a large degree, the physical properties of the oils, and hence their practical applications and commercial values. The particular FAs in the plant TAGs are not distributed randomly between the different *sn*-positions [28, 29].

## 4. Enzymes acting in lipid modifications

Oils and fats have belonged among the most important renewable materials for the chemical industry. Industrial oleochemistry has concentrated predominantly on reactions involving carboxylate functionalities of the fatty acids. Modern synthetic methods have been extensively applied to all types of natural FA derivatives for the selective functionalization of their alkyl chains [30]. Application of enzymes acting in lipid modifications displays increasing role among those methods, especially when the products are considered for application in pharmacology or in food industry [31].

Hydrolysis of plant oils has been an important reaction for the production of fatty acids and glycerol as a major source of surfactants and detergents. Another process of industrial importance has been the hydrolysis of vegetable oils to enrich them with FFAs [32–34], especially PUFAs, which have been of considerable pharmaceutical interest due to their biomedical properties. A replacement of the present chemical processes has been strongly required, because high temperature, high pressure and presence of inorganic catalysts are involved in the hydrolysis of oil [35]. The environmentally friendly and sustainable alternatives are enzyme-catalyzed processes. The mild temperature conditions required for enzymatic hydrolysis allow energy saving and lead to a product of improved quality (color and flavor) free of traces of inorganic catalysts. Lipases and other less frequently used enzymes acting in lipid modifications (lipoxygenases and phospholipases) can selectively lower activation energies, provide higher reaction specificities and enhance reaction rates in comparison to non-enzymatic reactions [36]. The enzyme-catalyzed hydrolysis can be performed in aqueous, organic or other non-conventional reaction media, e.g. supercritical fluids, ionic liquids and their combinations [37]. While organic solvents affect the nature of biocatalysis in several ways, including interaction with the essential hydration level of the enzyme, direct participation in the reaction mechanism, alteration of protein structure and its flexibility and alteration of the observed enzyme kinetics [38], supercritical fluids and ionic liquids represent environmentally friendly media for enzyme catalyzed processes [37, 39].

### 4.1. Lipases

**Scheme 2.**
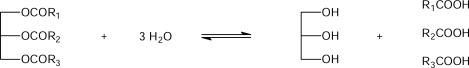
Lipase-catalyzed reversible process of a triacylglycerol hydrolysis and formation.

**Scheme 3.**
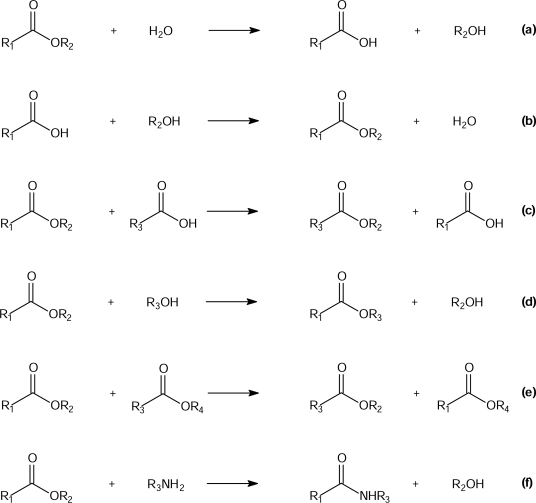
Processes catalyzed by lipases: (a) enzyme-catalyzed hydrolysis, (b) enzyme-catalyzed esterification, (c) enzyme-catalyzed transesterification by acidolysis, (d) enzyme-catalyzed transesterification by alcoholysis, (e) enzyme-catalyzed interesterification and (f) enzyme-catalyzed aminolysis.

Lipases (triacylglycerol hydrolases; E.C.3.1.1.3) belong to the family of serine hydrolases and can be found in animals, plants, fungi and bacteria [40–43]. They can also catalyze the formation of acylglycerols from FFAs and glycerol because the reaction they catalyze is reversible (Scheme 2). In the industrially developed countries, lipids present in the human diet mainly consist of triacylglycerols (TAGs), from 100 to about 150 g per day, i.e., 30% of each individual’s daily caloric intake. However, these TAG molecules cannot cross the intestinal barrier. A series of hydrolytic and absorption stages are necessary to produce the chemical energy resources present in the hydrocarbon chains of biologically usable TAGs. Lipases in the digestive tract play an important role in nutrition processes, both in humans and in higher animals [44]. Some lipases are able to control access to their active site. However, most of the lipases, which are used in laboratory investigations and/or in industrial production, are substrate tolerant enzymes, which accept a large variety of natural and synthetic substrates for biotransformation. Lipases do not require cofactors. They are often used in both, free or immobilized forms, and are commercially available, relatively inexpensive, and display relatively high stability. They act at the lipid-water interface and, therefore, they do not require water-soluble substrates. This function distinguishes lipases from other hydrolytic enzymes, and their efficiency in conducting transformations in organic solvents under mild conditions increases their importance as useful tools in organic synthesis [40–43,45–48]. Reactions in which lipases may be involved, both in nature and in laboratory or industrial application, are: (a) enzyme-catalyzed hydrolysis, (b) enzyme-catalyzed esterification, (c) enzyme-catalyzed transesterification by acidolysis, (d) enzyme-catalyzed transesterification by alcoholysis, (e) enzyme-catalyzed interesterification and (f) enzyme-catalyzed aminolysis (Scheme 3).

Lipase-catalyzed reactions are carried out under mild conditions: temperature lower than 70 °C, atmospheric pressure and with higher selectivity than their chemical counter parts. These enzymes catalyze the hydrolysis of glycerides at oil-water interfaces *in vivo*. In organic media with low water activity, they catalyze esterification [49] and interesterification of TAGs via alcoholysis, acidolysis, and transesterification [50–75]. Most of these works are kinetic studies on model reactions of acidolysis [53, 59, 60, 63–65] at lab-scale, and frequently in the presence of organic solvents [53–56, 63]. The bioproduction of TAGs enriched with ω-3 PUFAs has also been attempted via acidolysis [66–73]. In these systems, the recovery of the modified TAGs is rather difficult. Therefore, for industrial purposes, lipase-catalyzed transesterification-ester interchange, seems to be a more adequate route than acidolysis [51, 55–57, 74, 75]. The fats were obtained by transesterification of palm oil stearin with a concentrate of TAGs enriched with ω-3 PUFAs and soybean oil, catalyzed by a commercial immobilized *Candida antarctica* lipase (Novozyme 435) in solvent-free media.

Lipases occur widely in animals, plants and microorganisms [76]. One attractive feature of lipases is their specificity with respect to the glyceride position and fatty acid type, which could seldom be constructed by chemical catalysis [77]. Although a number of lipases, especially those in microorganisms, have been well characterized, knowledge of their structure-activity relationships remains sparse [78]. Lipase-catalyzed reactions offer several benefits over chemically-catalyzed reactions, such as milder operation conditions [79].

### 4.2. Lipase structure and catalytic ability

A mobile element, known as “lid”, consisting of either one or two short α-helices linked to the body of the lipase by flexible structural elements is present in most of lipases. In the open active site of lipases, the lid moves away making the binding site accessible to the substrate [80]. On the other hand, the lid needs an interface to be opened [81–85]. Three types of lipases could be identified according to their coordination-substrate site [86]: (a) Lipases corresponding to the *Rhizomucor* family including *Thermomyces* (formerly *Humicola*) *lanuginosa*, which have active sites and lids on the surface of the enzymes; (b) Lipases corresponding to the *Pseudomonas* and *Candida antarctica* family, which have active sites and funnel-like lids. *Candida antarctica* lipase B exhibits a very small lid and a funnel-like binding site; (c) Lipases corresponding to the *Candida rugosa* family, characterized by the presence of active sites at the ends of tunnels containing the lids in their external parts [87]. This peculiarity affects the coordination of the substrate because the reaction catalyzed by *R. miehei* is stimulated in either positions *sn*-1 or *sn*-3 – rather than in the position *sn*-2 – of the triglyceride, whereas in *C. rugosa* the serine, which is a part of the catalytic triad of the lipase, attacks all positions of the triglyceride [84].

### 4.3. Lipase-catalyzed hydrolysis under conventional conditions

Different lipases show different specificity, which is the cleavage of various bonds, chain length and structures of the cleaved fatty acids. Bottino *et al*. [88] noted that pancreatic lipase shows less activity towards icosapentaenoic acid and docosahexaenoic acid. They attributed the resistance to the location of a *Z*-configured double bond near the esterified carboxyl group or to the presence of several *Z*-configured double bonds in the chain placing the terminal methyl group in a position near the ester bond, thereby causing steric hindrance. Jensen *et al*. [89] reported that 15 triacylglycerols containing double bonds at various positions were hydrolyzed by pancreatic lipase. The substrates containing the Δ^2^ through Δ^7^ isomers of octadecaenoic acid were resistant to pancreatic lipolysis. The discrimination was greatest against the Δ^5^ isomer in the octadecaenoic acid series, when the double bond is beyond C(7). Moreover, *Geotrichum* lipase exhibits a high degree of discrimination in favor of acids having a *cis* double bond at the Δ9 position [89]. However, Lopez-Martinez *et al*. [90] reported the hydrolysis of borage seed oil containing γ-linolenic acid (6,9,12-octadecatrienoic acid) using two kinds of microbial lipases: (a) When employing *Candida cylindracea* lipase for catalyzing hydrolysis of the borage oil, γ-linolenic acid was accumulated in the non-hydrolyzed glyceride residue due to the lipase specificity towards γ-linolenic acid exhibiting Δ6 unsaturation; (b) With the *Chromobacterium viscosum* lipase, no accumulation of γ-linolenic acid was observed. Using this resistance to fatty acids, when fish oil was partially hydrolyzed by the microbial lipases produced by *C. cylindracea*, *A. niger* or *G. candidum*, the content of ω-3 polyunsaturated fatty acid in the remaining glycerides significantly increased [91, 92].

### 4.4. Lipase-catalyzed reactions under non-conventional conditions

Plant seed oils are one of the most important sustainable sources of FAs. Hydrolysis is the basic procedure of modification of natural triacylglycerols. Plant oils also contain certain quantities of additional natural products with pharmacological importance: vitamins, phytosterols, phytosteroids and other compounds. They are mainly isolated from oilseeds during the deodorization process within the volatile by-product known as deodorizer distillate at a quantity between 2 and 20 %, together with FFAs, mono-, di- and triacylglycerols [93, 94]. Modern non-conventional media (supercritical fluids, ionic liquids or their combination) are nowadays applied in obtaining FFAs from plant seed oils and modifying their structure by subsequent enzyme-catalyzed transformations [31, 95–97].

#### 4.4.1. Properties of supercritical carbon dioxide

**Figure 2.**
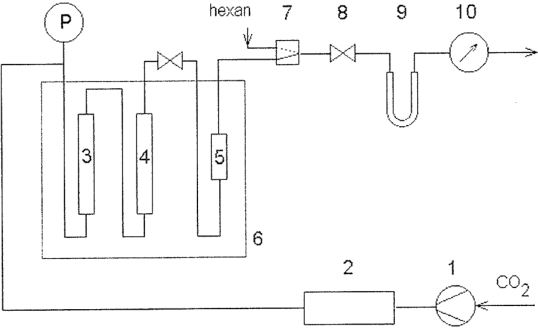
System for performing enzyme-catalyzed reactions in supercritical carbon dioxide: (1) compressor, (2) pressure control unit, (3) and (4) optional mixing or saturation units, (5) reactor, (6) oven, (7) rinsing valve, (8) micrometer valve, (9) trap, (10) gas meter.

Supercritical fluids have unique properties, which can be applied to a wide range of novel chemical processes [98–103]. Among them, supercritical carbon dioxide, defined as carbon dioxide above its critical point (t_c_ = 31.3 °C, P_c_ = 7.4 MPa), has the added benefits of an environmentally benign nature, non-flammability, low toxicity and ready availability, and it exhibits similar properties to organic solvents. It differs from ordinary solvents in having a combination of gas-like properties (i.e. low viscosities and high diffusivities which make them favorable for mass transfer) and liquid-like properties (i.e., solubilizing power) [104], which are tunable by the manipulation of pressure and temperature. Small variations in pressure or temperature lead to significant changes in density and density-dependent solvent properties such as dielectric constant, solubility parameters and partition coefficient, and render them more attractive as ‘green designer’ solvents and promising reaction media for environmentally benign chemical processes [104]. A scheme of the system used for extractions and enzyme-catalyzed processes operated under supercritical conditions is shown in Figure 2.

#### 4.4.2. Properties of ionic liquids

Ionic liquids are substances composed entirely of ions [105–107]. They are relatively polar solvents and promote the dissolution of a vast array of pharmaceutical intermediates and final drug molecules [108, 109]. The ability to modify the physico-chemical properties of these substances by simple structural modifications to the cations or changes in the anions increases the importance of ionic liquids in organic chemistry [105, 108, 110–112].

#### 4.4.3. Lipase-catalyzed hydrolysis in supercritical carbon dioxide

The application of supercritical carbon dioxide as a solvent in enzyme-catalyzed reactions has been a matter of considerable research because of its favorable transport properties, which accelerate mass-transfer-limited enzymatic reactions [113]. The inherent gas-like low viscosities and high diffusivities of supercritical fluids increase the rates of mass transfer of substrates to the enzyme. Conversely, the liquid-like densities of supercritical fluids result in higher dissolution compared to those observed for gases. Unlike the behavior of gases and liquids, the physical properties of supercritical fluids can be adjusted over a wide range by a relatively small change in pressure or temperature [114]. Moreover, it might be relevant to stress much lower expenses of solvent in the supercritical medium compared with those of conventional solvents [35]. Another advantage of the use of supercritical fluids (gases) as reaction media is the simple separation of oil and water in a continuously operated reactor on an industrial scale, due to their different solubility in these media. Since the solvent strength of a supercritical fluid can be varied by changing pressure and temperature, the solubility of substances can easily be regulated. It can be done continuously at the outlet of the reactor, allowing an integrated reaction and separation step, and thus simplifying the downstream processing and recycling of the solvent [35].

Examples of industrially important hydrolytic processes catalyzed by lipases are productions of soaps [43,115], fatty alcohols [43] and monoacylglycerols [116,117]. Knez *et al*. [118] studied the thermodynamic and kinetic properties of the immobilized lipase from *Aspergillus niger* (Lipolase 100T), and used this enzyme for catalysis of hydrolysis of sunflower oil in supercritical carbon dioxide. Employing a high-pressure continuous flat-shape membrane reactor, they achieved maximum conversion after 1 hour of the reaction at 50 °C, 90 MPa, and at a flow rate for substrates of 0.1 mL/min, improved during following investigation [119, 120].

We have studied blackcurrant seed oil, which is rich in α- and γ-linolenic acid, which the human organism is unable to synthesize *de novo* [121]. To be able to isolate α- and γ-linolenic acid from blackcurrant seed oil, we used Lipozyme, a lipase from *Mucor miehei*, immobilized on macroporous anionic resin [122,123]. The reaction was performed in a continuous flow reactor at 10–28 MPa and 30–50 °C with carbon dioxide saturated with oil and water (55 – 100%) flowing up through the enzyme bed. Analysis of product composition indicated unfavorable hydrodynamics with significant mixing in the reactor when solvent interstitial velocity was lower than 4 cm/min, while above this velocity value the flow pattern was near to plug flow. Lipase stability was very good with no activity reduction observed during a long-term experiment. The reaction rate was a function of the ratio of enzyme load to solvent volumetric flow rate. A complete hydrolysis of oil was achieved in the experiments carried out with the enzyme load of 0.8 g and CO_2_ flow rate of 0.4–0.9 g/min. The effects of pressure (10–25 MPa) and temperature (30–40 °C) on the reaction rate were small, and the effects of CO_2_ saturation with water and of enzyme distribution in the reactor were negligible. Lipozyme displayed specificity towards linolenic acids; the release of α-linolenic acid was faster and that of γ-linolenic acid slower than the release of other constituent acids present in blackcurrant oil. The experimental system was also studied and analyzed by means of HPLC-NMR hyphenated technique [124].

### 4.5. Lipase-catalyzed esterification in organic solvents

Many lipases catalyze esterification reactions in organic solvents. TAG synthesis from DHA ethyl ester has been performed using the lipase from *Candida antarctica* immobilized on macroporous acrylic resin in a yield > 95% at 50°C for 23 h [125]. Not only plants are rich sources of PUFAs. For instance, the esterification of glycerol and ω-3 PUFAs from cod liver oil was studied using Lipozyme IM from *Mucor miehei*, Novozym 435 from *C. antarctica* and lipase PS from *Pseudomonas* [126]. Novozym 435 has been proven to be highly active. Medina et al. [127] studied the production of commercially viable EPA-rich TAG from cod liver oil and the microalga *P. tricornutum* and TAG rich in EPA and AA from the microalga *P. cruentum* by esterification of these PUFAs with glycerol by lipase-catalyzed reactions.

### 4.6. Lipase-catalyzed transesterification and acidolysis under conventional conditions

Enzyme-catalyzed transesterification reactions have been employed for modification of fats and oils. The process involves rearrangement of fatty acid moieties within triacylglycerol molecules, with consequent improvement of the physico-chemical characteristics of fats and oils. Lipases have been used as biocatalysts for the modification of fatty acid profile of vegetable and marine oils to produce structured lipids, which are currently manufactured for their potential clinical benefits and for food applications. These nutraceutical lipids may provide specific fatty acids with particular properties desirable in specific disease or pathological conditions. A number of studies have focused on obtaining TAG having a combination of ω-3 and ω-6 PUFAs.

Senanayake and Shahidi [13] tested nonspecific *C. antarctica*, *sn*-1,3 specific *M. miehei*, and nonspecific *Pseudomonas* sp., which were able to catalyze incorporation of EPA and DHA in the borage and evening primrose oils to various extents. Among the lipases tested, *Pseudomonas* sp. gave the highest degree of EPA and DHA incorporation in both oils (32.6 and 32.3% after 36 h, in borage and evening primrose oils, respectively) followed by *M. miehei* and *C. antarctica*. The most effective lipase was selected for subsequent experiments to determine optimal acidolysis conditions. Previously, Akoh and Moussata [128] used two immobilized lipases, nonspecific SP435 from *C. antarctica* and *sn*-1,3 specific IM60 from *Rhizomucor miehei* as biocatalysts for restructuring of borage oil to incorporate EPA and capric acid (10:0) with free fatty acids as acyl donors. They obtained higher incorporation of EPA (10.2%) and capric acid (26.3%) using IM60 lipase, compared to 8.8 and 15.5%, respectively, when SP435 lipase was used. The ability of immobilized lipases IM60 from *M. miehei* and SP435 from *C. antarctica* to modify the fatty acid composition of selected vegetable oils (canola, peanut, and soybean oils) by incorporation of ω-3 PUFAs into the vegetable oils was studied [69] by using FFAs and FAEEs of EPA and DHA as acyl donors. Using free EPA as acyl donor, IM60 gave higher incorporation of EPA than SP435. However, with FAEEs of EPA and DHA used as acyl donors, SP435 gave higher incorporation of EPA and DHA than IM60.

### 4.7. Structured lipids

Structured lipids (SLs) are triacylglycerols or phospholipids in which FAs can be found in specific locations in the glycerol backbone and are produced using chemical or enzymatic processes. SLs are new generation fats or oils with medical, nutraceutical and food applications. Lipids can be restructured to meet EFA requirements or to incorporate specific FAs of interest into specific locations of the glycerol backbone of TAG. SL may offer the most efficient means of delivering target FAs for nutritive or therapeutic purposes as well as to alleviate specific disease and metabolic conditions.

The constituent FAs and their locations in the glycerol backbone determine the functional and physical features, the metabolic fate, and the health benefits of SLs. Therefore, designing SLs with selected FAs at specific locations in the TAGs for medicinal applications has attracted much attention. The position of FA in the TAG molecules (*sn*-1, *sn*-2, and *sn*-3) has a significant impact on their metabolism in the body. In general, FAs at the terminal positions of TAG (*sn*-1 and *sn*-3) are hydrolyzed by pancreatic lipase and absorbed, while those at the *sn*-2 position of TAG remain unchanged and are used in the synthesis of a new TAG. For example, it may be desirable to develop a SL containing PUFAs at the *sn*-2 position with medium-chain fatty acids (MCFAs) at the *sn*-1,3 positions for patients with mal-digestion as well as cystic fibrosis. A SL containing MCFA and linoleic acid is more efficient in cystic fibrosis patients than safflower oil, which has about twice as much of linoleic acid [129]. SLs have many industrial applications and have recently attracted the attention of food manufacturers for production of low-caloric lipids that are characterized by a mixture of short-chain fatty acids (SCFAs) and/or MCFAs and LCFAs in the same glycerol moiety. Increasing interest in such products stems from the fact that they contain 5 - 7 kcal/g energy compared to 9 kcal/g for usual fats and oils; this is because of the lower caloric content of SCFA or MCFA compared to LCFA. Reduced-calorie specialty lipids are intended for use in baking chips, dips, coatings, bakery and dairy products, or as a cocoa butter equivalent.

Over the past two decades several research groups have successfully incorporated MCFAs (caprylic acid or capric acid) into fish and marine oils containing PUFAs [72,128–133] into borage oil rich in γ-linolenic acid [128, 130, 133] and into single-cell oils [134–137]. SLs may be produced by incorporation of selected FAs into an oil. The degree of reactivity of different FAs may vary in different systems due to factors such as the lipase type, water activity, and other conditions [138]. Many lipases have been shown to be more selective toward C_18_ FA with higher degrees of unsaturation in esterification and interesterification reactions (C18:0 < C18:1 < C18:2) [139]. Yang *et al*. [138] compared incorporation of linoleic and conjugated linoleic (CLA) acids into tristearin (SSS) in a solvent-free system at 60 °C using 5% Lipozyme RM IM from *Rhizomucor miehei*. Incorporation of LA into SSS was higher than that of CLA and suggested that the rigidity of CLA might have been responsible for this observation [138]. Tsuzuki [140] screened ten lipases for their ability to catalyze acidolysis of triolein with SCFAs (C2:0, C3:0, and C4:0) in organic solvents. Lipase from *Aspergillus oryzae* afforded the highest yields of products in the reaction of triolein with C2:0, C3:0, and C4:0. The results of the study indicated that as the chain length decreased, the degree of incorporation of SCFAs into triolein increased. Paez *et al*. [141] reported that incorporation of caprylic acid (C8:0) into triolein was favored compared with that of oleic acid. Again chain length of the FA might play a role in the observed trends. The synthesis of a modified oil via acidolysis of trilinolein (tri C18:2) with C8:0, using Lipozyme IM-60 as a biocatalyst has been reported [142]. Lipozyme IM-60 was found to be more effective for incorporation of a LCFA than a MCFA. A synthesis of SL by interesterification of trilinolein and tricaproin with *sn*-1,3-specific (IM 60) and nonspecific (SP 435) lipases was reported [143]. In general, it was found incorporation of selected LCFAs into TAGs (e.g. tristearin or triolein) may be affected by many factors, including chain length, number of double bonds, and the location and geometry of the double bonds as well as reaction conditions and reactivity and specificity of lipases employed. LA was more reactive than CLA due to the rigidity of the latter and/or specificity of the enzymes [144]. EPA was more reactive than DHA, due to the structural differences between the two (number of double bonds, chain length) [145]. Lipases, Novozyme-435 enzyme from *C. antarctica* and AY-30 from *Candida rugosa*, might be considered as promising biocatalysts for acidolysis of tristearin and selected LCFAs [146]. The high percent incorporation of FA into tristearin using lipase from *C. antarctica* or *C. rugosa* might be due to the experimental conditions employed in the study which were suitable for these two enzymes [145]. Hamam and Shahidi [145] reported the acidolysis of tristearin and triolein [144] with LCFAs. In the study, they examined the effect of the chain length, number of double bonds, and the location and geometry of double bonds on the incorporation of selected FA into tri C18:2 and tri C18:3. Several studies have dealt with the synthesis of SL enriched in PUFAs using a one-step lipase catalyzed synthesis [137,147,148]. However, this synthesis required long reaction times and achieved only low yields of the desired products. The two-step reaction was recently suggested as an effective method for the SL synthesis resulting in higher yields and purer products than the conventional one-step reaction [149, 150]. In the first-step, 2-MAG are produced by alcoholysis of a triglyceride with ethanol using a 1,3-regiospecific lipase. The 2-MAG thus obtained can subsequently be esterified with suitable FAs.

#### 4.7.1. Lipase-catalyzed esterification in supercritical carbon dioxide

Lipase-catalyzed production of various types of esters, including SLs, has increased tremendously within the recent years. Esters are mainly present in oils, fats and natural polymers, and they are useful intermediates or target products in chemical industry. Acylglycerol and aliphatic ester synthesis by lipases was described [151, 152], and proved the occurrence of reverse reaction. Lipases from *Aspergillus niger*, *Rhizopus delemar*, *Geotrichum candidum* and *Penicillium cyclopium* catalyze the synthesis of oleic acid esters derived from a number of primary alcohols. Among these lipases, only that from *G. candidum* was able to catalyze the synthesis of esters of secondary alcohols. It can be achieved either by reaction between FFA and an alcohol or by ester exchange or transesterification, which includes alcoholysis, acidolysis and interesterification [45].

Lipase-catalyzed esterification reactions have been applied in a production of different types of important esters: (a) esters of short-chain alcohols and short-chain fatty acids (aroma compounds; [45]), (b) esters of short-chain alcohols and long-chain fatty acids (oleochemicals, e.g. lubricants, diesel fuel, and antistatic reagents; [45]), (c) esters of polyhydroxy alcohols (glycerol, alcoholic sugars, carbohydrates etc.) and long-chain fatty acids, which are generally called emulsifiers or surfactants (important products for application in food and pharmaceutical industries; [45]).

Lipases have often been used for treatment and modification of oils and fats [45]. Many lipases exhibit *sn*-1,3 specificity and may be used for regioselective (inter)esterification of natural triacylglycerols. The acyl migration observed from the *sn*-2 to the *sn*-1 or *sn*-3 positions must be suppressed or eliminated, wherever possible. By partial or total exchange of fatty acyls in TAGs of given origin it is possible to modify physico-chemical properties and also nutritional value of the starting natural oil or fat [45]. Three types of triacylglycerol modification are commercially and pharmacologically important: (a) production of cocoa butter equivalents, (b) production of fats with improved spreadability and (c) production of highly digestive triacylglycerols [43, 45].

(a) Cocoa butter equivalent: The main producers of cocoa butter are tropical countries, mainly Kenya and Malaysia. Since the melting point of cocoa butter is around human body temperature (37 °C), it is well suited as a matrix for suppositories. The main application is in the production of chocolates, where the rapid melting conveys a desirable feeling. The predominant TAGs of cocoa butter are compounds bearing oleic acid in the *sn*-2 position and stearic and palmitic acids in the *sn*-1 and *sn*-3 positions (SOS and SOP). Cocoa butter equivalents can be prepared either chemically or by lipase catalysis through the interesterification of suitable natural triacylglycerols, for example, the middle fraction of palm oil (POP) or sunflower oil with a high content of oleic acid (high-oleic sunflower oil; OOO) with stearic acid or tristearin (SSS) [43,153]. The primary hydroxyl groups of glycerol in positions *sn*-1 and *sn*-3 are more reactive than the secondary hydroxyl group in the *sn*-2 position, and TAGs of the SOP or SOS type are predominantly formed.

(b) Fats with improved spreadability: The melting point of any oil can be modulated by the degree of catalytic hydrogenation of double bonds in unsaturated FAs, as it is done on a large scale for the preparation of margarines and shortenings from plant oils. Alternatively, the desired melting point can be achieved through interesterification of suitable triglyceride mixtures with the use of *sn*-1,3 specific lipases [43].

(c) Highly digestive triacylglycerols: The absorption of TAGs from the small intestine strongly depends on their structure. TAGs containing palmitic acid are well adsorbed only when this FA is located in the *sn*-2 position, like in human milk [154]. A commercial product of this type (OPO) is a diet additive for premature infants. It is prepared by interesterification of tripalmitin with oleic acid with use of immobilized *Rhizomucor miehei* lipase. Alternatively, TAGs of the MLM type, with long-chain saturated or unsaturated FAs (L) in the *sn*-2 position and medium-chain fatty acids (M) in the positions *sn*-1 and *sn*-3, provide a rapid delivery of energy through enhanced hydrolysis and resorption; pancreatic lipase preferentially hydrolyzes medium-chain triacylglycerols, and the resulting MAGs are efficiently absorbed from the large intestine. Several products of this type are commercially available, which contain PUFAs (EFAs), and have shown beneficial effects against cardiovascular and inflammatory diseases. Functional TAGs of this composition are preferentially prepared by means of *sn*-1,3 specific lipases during an interesterification process starting from highly unsaturated TAGs [43].

#### 4.7.2. Other types of lipase-catalyzed reactions in supercritical carbon dioxide

The utilization of supercritical carbon dioxide as a reaction medium confers many advantages, among which environmental compatibility, zero chemical residues in the synthesized product, and considerable processing flexibility are the most important [155]. When lipase is used for catalyzing a synthetic procedure in supercritical carbon dioxide, the process is particularly applicable to producing additives that can be incorporated directly into food formulations [45,156–161]. A synthesis of simple esters [162] was reported, transesterifications to make methyl esters [156] were conducted, a glycerolysis process [156] was studied, and randomization of fats/oils [156] in supercritical carbon dioxide was performed using *Candida antarctica* lipase (Novozym 435; [163]). High quantitative yields of transesterifications in making methyl esters have permitted application of the lipase reaction in supercritical carbon dioxide as an analytical method for quantifying fat levels in food products, required under new food nutritional labeling guidelines [164]. A Novozym 435-catalyzed transesterification has been utilized as the initial step in a two-stage synthesis conducted under supercritical fluid conditions to produce fatty alcohols directly from vegetable oils [165].

An interesting study has been performed with lipase-catalyzed esterification of stearic acid with ethanol, and subsequent hydrolysis of ethyl stearate under the conditions set near to the critical point in supercritical carbon dioxide (p = 6 to 20 MPa, t = 35 to 60 °C) [166], resulting in an observation of the esterification rate of stearic acid increasing near the critical point and keeping the increase steady with increasing the pressure, and reflecting the increasing solubility of stearic acid in supercritical medium. The hydrolysis rate of ethyl stearate showed its maximum at a pressure near the critical point, and it was dependent on the initial concentration of ethyl stearate in the system. When the reaction was performed with an initial ester concentration below the solubility limit in supercritical carbon dioxide, the authors observed the maximum pressure shifted along the extended line of the gas-liquid equilibrium in the supercritical region in the pressure-temperature phase plan. This finding seems to be related to the singular behavior of some properties of supercritical carbon dioxide along this line reported in the literature [167].

Myristic acid was esterified by ethanol using a hog pancreas lipase in supercritical carbon dioxide in 37 % yield, while a similar enzyme-catalyzed reaction performed in acetonitrile yielded only 4 % of the required product [168].

Immobilized lipases from *Thermomyces* (*Humicola*) *lanuginosa* and *Candida antarctica* (lipase B) were employed for modification (alcoholysis and glyceride synthesis) of cod liver oil with ethanol in supercritical carbon dioxide [169]; fish oil is rich in important PUFAs, like many plant and seed oils, and unlike animal fats. Long-chain FA esters with fatty alcohols are useful functional molecules in pharmaceutical, cosmetic and lubricant industry. Knez *et al*. [170, 171] used lipase from *Rhizomucor miehei* (Lipozyme RM IM) as catalyst for production of 1-octyl oleate in supercritical carbon dioxide, and studied the influence of parameter changes to the conversion rate in bench-scale packed-bed reactor. The authors got 93 % maximum yield of the product at a substrate flow rate of 18 mL/h and the CO_2_ flow rate of 210 mL/h, and the immobilized enzyme was able to act as catalyst up to 50 days. They proved a possibility of using such a system for continuous production process.

Modification of castor oil in supercritical carbon dioxide may be an example of *C. antarctica* immobilized lipase B catalyzing interesterification between castor oil TAGs and methyl oleate in 90 % yield within 5 hours [172]. Even naturally occurring aliphatic amides, like capsaicin (8-methyl-*N*-vanillyl-6-nonenamide), a compound from the red pepper seeds, promoting blood circulation, are compounds of interest for pharmacology. It is relatively hardly available in broad scale. Palmitoyl vanillylamide, one of its analogs, can be synthesized through amidation by immobilized *Mucor miehei* lipase in supercritical carbon dioxide under optimized conditions (50 °C, 17 MPa, pH 8, 8 hours) from vanillylamide hydrochloride and palmitic anhydride at a molar ratio 5 / 15, and enzyme concentration of 0.5 % (w/w) [173].

The course of the lipase-catalyzed reactions in supercritical carbon dioxide is always affected by the presence of moisture. Continuous acidolysis of triolein and stearic acid was studied [174] using the moist immobilized lipase in supercritical carbon dioxide in a large scale process at 50 °C, 16.9 MPa and adsorbed water concentration of 2 % (w/w). The production rate was about 0.03 mmol/h per each 1 g of the immobilized enzyme.

The activity of several lipases was studied in subsequent enzymic transformation processes, after having used them as biocatalysts of blackcurrant (*Ribes nigrum*) oil hydrolysis in supercritical carbon dioxide. The subsequent reactions were performed at 40 °C and 15 MPa in a continuous flow reactor. The most remarkable increase of enzyme activity was observed with a lipase from *Rhizopus arrhizus* and with Lipozyme (lipase from *Mucor miehei* immobilized on macroporous resin) [175].

### 4.8. Separation and lipase-catalyzed modifications of phytosterols and related minor compounds

Increasing importance of nutritional and pharmaceutical components (phytosterols, vitamins etc.) of plant oils has resulted in developing new sustainable processing methods [176]. Supercritical carbon dioxide extraction and fractionation techniques have been positively examined as alternative methods of obtaining plant and seed oils with high purity and quality [176, 177], and minor components of plant and seed oils (vitamins, phytosterols, isoprenes etc.) can also be separated. Canadian canola oil is one of the most known and most commercialized oils. Value-added processing of canola oil deodorizer distillate (concentrated mixture of phytosterols and tocopherols) has benefited the canola oil processing industry in Canada [178].

Synthesis of esters derived from phytosterols or other steroid alcohols and FAs is of great importance, due to their recent recognition and application in the food industry as cholesterol-lowering agents. Several enzymes were screened as catalysts, and optimal conditions were determined for the reaction between various FAs and sterols / phytosterols (e.g., cholesterol, sitostanol etc.) in supercritical carbon dioxide [156, 179]. Using an analytical supercritical fluid extraction unit, the lipase derived from *Burkholderia cepacia*, Chirazyme L-1, was appointed to be the most selective for facilitating the desired reactions [180]. FAs C_8_–C_18_, a pressure range of 20.7 – 31 MPa, a temperature range of 40 – 60 °C, along with variable flow rates, and initial static hold times were used to evaluate the feasibility of the above reaction. The yield of the cholesterol esters, as measured by supercritical fluid chromatography, ranged from 90 % for caprylic acid to 99 % for palmitic acid, while the corresponding reaction between sitostanol and the same FAs produced yields of 92 % for caprylic acid and 99 % for palmitic acid, respectively [156]. The extraction apparatus was modified to provide a continuous flow of the reagent FA and phytosterol or other steroid alcohols over the enzyme bed, thereby allowing continuous production of the desired esters, which averaged a 99% yield under optimal conditions [156].

### 4.9. Lipase-catalyzed reactions in ionic liquid / supercritical carbon dioxide biphasic systems

A synthesis of butyl esters (propanoate, laurate etc.) in a recirculating bioreactor using ionic liquid / supercritical carbon dioxide biphasic systems at 50 °C and 8 MPa was described as an example of application of biphasic systems [181,182], in which α-alumina microporous membranes with immobilized *Candida antarctica* lipase B were coated with four different ionic liquids based on 1-n-alkyl-3-imidazolium cations and hexafluorophosphate and bis{(trifluoromethyl)sulfonyl}imide anions. Selectivity increased up to > 99.5 % when the ionic liquid / supercritical carbon dioxide biphasic system was used rather than supercritical carbon dioxide alone (at room temperature). The activity in room temperature ionic liquid / supercritical carbon dioxide biphasic systems depends on the effect of the ionic liquid media on the enzyme and the diffusion limitations across the ionic liquid layer around the biocatalyst.

## 5. Conclusions: Importance of the reaction media for sustainability

Extraction of compounds from natural sources has been the most widely studied application of supercritical fluids with several hundreds of published scientific papers (cf. review [39]). Supercritical fluid extraction has immediate advantages over traditional extraction techniques: it is a flexible process due to the possibility of continuous modulation of the solvent power / selectivity of the supercritical fluid, allows the elimination of polluting organic solvents and of the expensive post-processing of the extracts for solvent elimination. Supercritical carbon dioxide, a ‘green’ and sustainable medium, is nowadays the most frequently used supercritical fluid for productions in nutraceutic, cosmetic and pharmaceutical industry.

Supercritical fluid extractions, fractionations of the extracts, and their enzyme-catalyzed modifications have been developed and have expanded tremendously in many fields of products. This approach is also scientifically challenging but also necessary to find applications that are industrially competitive with the traditional processes based on cheaper technologies and plants. In some cases, the extraction problems proved to be very complex and as a consequence more evolved process schemes (multistep extractions, continuous solid processing, multistage separations, co-solvent or ionic liquid applications) have been adopted to overcome these problems. However, a large quantity of work is still required. Nevertheless, numbers of reviews have recently appeared which have shown perspectives of application of supercritical fluid technology in many areas of research and industrial production [39, 45–47, 108, 109, 183–185].
